# High-Performance Liquid Chromatographic Method for Determination of Phenytoin in Rabbits Receiving Sildenafil

**DOI:** 10.4137/aci.s658

**Published:** 2008-04-18

**Authors:** Alaa Khedr, Mohamed Moustafa, Ashraf B. Abdel-Naim, Abdulrahman Alahdal, Hisham Mosli

**Affiliations:** Faculty of Pharmacy, King Abdulaziz University, Jeddah, Saudi Arabia

**Keywords:** phenytoin, sildenafil, rabbit plasma, chromatography

## Abstract

A validated high-performance liquid chromatographic (HPLC) method for determination of phenytoin (PHN), para-hydroxy metabolite of phenytoin (POH) and sildenafil (SIL) in rabbit plasma is described. The method is based on extraction on Sep-Pak C18 solid support using ethyl acetate and ether as eluents and monitoring at 220 nm. The extracted samples were analyzed by HPLC using Agilent Zorbax Extended C_18_ column (150 mm × 4.6 mm internal diameter) and isocratic elution with a mobile phase consist of 29% acetonitrile and 71% sodium acetate solution (0.02 M, pH 4.6). The method was fully validated for linearity and range, selectivity, precision, stability, recovery, and robustness. The linearity of the method was in the range of 0.15 to 39 μg /ml for PHN and 0.15 to 33 μg/ml for both POH and SIL. Limits of detection (LOD) of PHN, POH, and SIL were 0.15 ± 0.01, 0.15 ± 0.01, and 0.15 ± 0.01 μg/ml, respectively. The % recovery of PHN, POH, and SIL from rabbit plasma were, 101.88 ± 0.12, 99.16 ± 0.25, and 99.49 ± 0.33, respectively. The method was applied on plasma collected from rabbits at different time intervals after receiving 30 mg/kg PHN-Na with (and without) 8 mg/kg SIL citrate.

Phenytoin (5,5-Diphenylhydantoin, PHN) is one of the most commonly prescribed anticonvulsant drugs in the treatment of epilepsy (Fig. 1) (Leduc, 2002). Unfortunately, PHN has narrow therapeutic window, and careful monitoring of the drug-plasma level is necessary during therapy to avoid undesirable effects (Leduc, 2002). While 10% of healthy men had sexual dysfunctions, male epilepsy patients experience sexual problems in 40%–70% of the cases (Bone and Janszky, 2006; Stimmel and Gutierrez, 2006; Montouris and Morris, 2005; and Smaldone et al. 2004). Sildenafil citrate (*Viagra*®) is widely used as prescribed or non-prescribed agent for management of erectile dysfunction (Bone and Janszky, 2006). Assessment of potential pharmacokinetic interaction PHN and SIL awaits evaluation (Stimmel and Gutierrez, 2006). Therefore, the present work is designed to develop an accurate and precise analytical method to determine PHN in plasma in case of concomitant administration of SIL. The developed method is useful to explore the impact of sildenafil citrate administration on the pharmacokinetic behavior of PHN in human. Many analytical methods have been reported for the analysis of PHN and its metabolites (Bereczki et al. 2001; Guan at al. 2000; and Maya et al. 1992) and PHN in combination with other antiepileptic drugs in plasma (Vermeij and Edelbroek, 2007; Bugamelli et al. 2002; and Bhatti et al. 1998). Frequently published methods were included; high performance liquid chromatography (Vermeij and Edelbroek, 2007; Bugamelli et al. 2002; and Bhatti et al. 1998), thin layer chromatography (Simon et al. 1971, and Rao and Mclennon, 1977), gas chromatography (Queiroz et al. 2002), fluorescence polarization immunoassay (Lu-Steffes et al. 1982), and spectrophotometry (Rezaei et al. 2005). The extraction of PHN has been described by many publications which include solid-phase extraction (SPE) on C18 column (Guan at al. 2000; Vermeij and Edelbroek, 2007; and Bugamelli et al. 2002). SPE on molecularly imprinted solid-phase (Bereczki et al. 2001) or liquid-liquid extraction (Guan at al. 2000). The percentage recoveries of PHN from plasma were varied from 60%–94% using SPE. On the other hand, many chromatographic methods have been reported for the determination of SIL and its major metabolite in plasma using different detection methods (Ghazawi et al. 2007; Wang et al. 2005; and Cho et al. 2003). Because of the big differences in the physiochemical properties, metabolic fat, and dosing of both, PHN and SIL (Moffat et al. 2004), it was necessary to develop an HPLC method capable for the analysis of this combination in one run. In addition to the need of developing a suitable extraction procedure to recover both PHN and SIL in high yield.

## Experimental Section

### Chemicals and reagents

Phenytoin sodium was purchased from Spectrum, Gardena, NJ, U.S.A. Phenobarbitone sodium (In St) was purchased from EVANS Medical LTD, Liverpool, England. Sildenafil citrate was obtained as gift from Sanofi, Jeddah, Kingdom of Saudi Arabia. Phenytoin para-hydroxy metabolite (POH) was synthesized in our lab and checked for purity by spectral and chromatographic methods (Melton and Henze, 1947; and Claesen et al. 1982). All solvents were of HPLC or spectroscopic grade. All other materials were of Analar grade (sodium acetate, sodium hydroxide, formic acid, acetic acid 100%, diethylamine, and trifluoroacetic acid). Dosing, feeding and sample collection were carried in our toxicology laboratory. Rabbit plasma samples were kept at −20 °C until use.

### Equipment

The HPLC system consisted of an Alliance Waters separations module 2695, waters 2996 Photodiode array detector (Milford, MA, U.S.A.) set to 220 nm. Column heater was set to temp 25 ± 2 °C. HPLC system control and data processing was performed by Empower software (Build 1154, Waters). Screw capped V-shaped vials (capacity, 300-μl) were used (Alltech, GmbH, Unterhaching, Germany). Digital micro-transfer pipettes 5–250 μl (Brand, Wertheim, Germany). Sep-Pak C_18_ extraction columns were purchased from Waters Corporation, Milford, MA, U.S.A. (part #: WAT051910).

### Chromatographic conditions

Analytes were separated on Agilent Zorbax Extend-C_18_, 150 × 4.6 mm, 80Å, 5 μm and protected with pre-column: Agilent Zorbax Extend-C18, 4.6 × 12.5 mm, 80Å, 5 um (Agilent Technologies, Palo Alto, CA, U.S.A.). The mobile phase was composed of 29% acetonitrile and 71% sodium acetate (0.02 M adjusted to pH 4.6 with acetic acid) and pumped at a flow rate of 1.0 ml/min. The analytical column and pre-column were kept at 25 ± 2 ºC. The analytical column was washed with acetonitrile for 20 minutes after each five runs.

### Standard and calibration solutions

Standard stock solutions of PHN-sodium and SIL citrate were prepared separately in water; however, a standard stock solution of POH was prepared in acetonitrile, to give concentrations of 2.6, 1.2 and 1.5 μg/μl, respectively. The internal standard (In St) solution was prepared by dissolving 25 mg phenobarbitone sodium in 10 ml water, and 1 ml from this solution was further diluted to 10 ml with acetonitrile to give a concentration of 250 ng/μl. Appropriate dilutions in acetonitrile were prepared from each stock solution to obtain a mixtures of the calibration standards containing PHN sodium, POH and SIL citrate spanning the range of 1.1–78.0, 1.0–66.0 and 1.0–68.0 ng/μl, respectively. A volume of 100-μl from four strengths of the calibration standard solutions, covering all range, were spiked in blank rabbit plasma to prepare the quality control (QC) samples. The QC samples were divided in small aliquots and stored at −20 °C until use. A sample volume of 200 μl of each QC sample was extracted and analyzed at time intervals of; 0, 10 and 30 days. All plasma extracts were spiked with 50 μl internal standards solution (250 ng/μl phenobarbitone sodium in acetonitrile) before the step of drying.

### Calibration curve in plasma

The Sep-Pak C18 cartridges were fitted in a 25-ml plastic syringe. About 3-ml volume of water purged throughout the column, and the air was forced to pass through the column to remove any unbound water. A volume of 200 μl plasma was transferred to the side bottom of the syringe, spiked with 100 μl of each calibration standard solution mixture basified with 100 μl of diethylamine (5% in acetonitrile). The sample was thoroughly swirled and forced onto column using syringe piston. The Sep-Pak columns were detached from the syringe and a compressed air was allowed to pass through the column for 30 seconds in the elution direction to remove extra plasma liquid. The Sep-Pak columns were then attached again to the syringe and a volume of 10-ml of ethylacetate was poured into the syringe column and allowed to flow through the Sep-Pak column with approximate flow rate of 2 ml/min. A volume of 100 μl of trifluoroacetic acid (5% in acetonitrile) was added onto the extraction column, and then a volume of 10 ml of diethyl ether was added and allowed to flow through the Sep-Pak column with approximate flow rate of 2 ml/min. The extracts were combined in test tube containing 50 μl of In St and dried with gentle stream of nitrogen gas at 40 ºC. The residues were reconstituted with 150 μl acetonitrile, vortexed for 0.5 min, 150 μl sodium acetate (0.02 M, pH 4.6), vortexed for 0.5 min, and transferred to 300-μl autosampler vials with Pasteur pipette. A volume of 50 μl was injected for HPLC analysis. The generated chromatograms were recorded and integrated with Empower WATERS software. The calibration curves corresponding to POH, SIL and PHN were drawn to calculate regression coefficient, slope, and intercept. The percentages of peak areas ration of the corresponding drug to In St were plotted versus concentrations in ng/μl.

### Drug recovery from plasma

The Sep-Pak C18 cartridges were fitted in a 25-ml plastic syringe. A volume of 200 μl plasma was transferred and mixed with, 100 μl standard solution mixture containing PHN, POH, and SIL (78, 66 and 68 ng/μl, respectively). Then the sample was extracted as described under title calibration curve in plasma using phenobarbitone sodium as internal standard. The same procedures were repeated using plasma spiked with PHN, POH and SIL (1.1 to 33.0 μg/ml, from each). The procedures were repeated three times for each concentration level.

### Animal treatment and sample preparation

Nine Male Albino-rabbits were randomly selected for this study. The average weight of rabbits was ranged from 1.0 to 1.5 kg. The rabbits were kept under standard feeding and housing conditions along the experiment. A dose of 30 mg/kg of phenytoin sodium (dissolved in 4 ml sterile water) was given intra-peritoneal once daily (od) for seven days. On day 7, blood samples were collected at times 0, 0.1, 1, 2, 3, 4, 6, 12 and 24 hr after the 7th dose. From day 8 to 14, PHN sodium was co-administered with oral SIL citrate, 8 mg/kg, od. On day 14, blood samples were collected at the same time points. Blood samples were kept on citric acid in a refrigerator at −20 °C till the time of analysis. Samples were centrifuged for 10 minutes at 15000 rpm; plasma was harvested and immediately frozen at −20 °C until use.

### Determination of phenytoin in rabbit plasma samples

The Sep-Pak C18 cartridges were fitted in a 25-ml plastic syringe. About 3-ml volume of water purged throughout the column, and the air was forced to pass through the column to remove any unbound water. A volume of 200 μl plasma was transferred to the side bottom of the syringe and basified with 100 μl of diethylamine (5% in acetonitrile) and extracted as described under title calibration curve in plasma using phenobarbitone sodium as internal standard. A volume of 50 μl was injected for HPLC analysis. The sample content was calculated from the calibration curve considering that the% recovery is about 100%.

### Calculations

The amount of PHN was calculated from the corresponding calibration curve as ng/μl (of the final injected solution) and multiplied by 1.5 to get the amount as μg/ml of plasma. The para-hydroxymetabolite of PHN was not monitored because it is predominantly excreted as glucuronide conjugate (Moffat et al. 2004).

## Results and Discussion

### Chromatographic variables

The best chromatographic conditions achieved are described at the experimental part. Enough separation between all drugs investigated with acceptable chromatographic performance parameters was obtained (retention time, capacity factor, resolution, tailing factor, theoretical plates, and selectivity coefficient). The retention time of SIL peak was critically affected by the variation of pH than In ST, PHN and POH. At pH 3.0 ± 0.6 an overlap between SIL and POH was observed. Also, at pH 3.0 ± 0.6 band broadening of SIL was observed. Bad resolution with band broadening of In St, PHN and POH with more retarded SIL peak if sodium acetate solution (71%, pH 4.6) replaced with formic acid (0.05% adjusted to pH 4.6 with 1M NaOH). Also, bad resolution with band broadening of all peaks was observed upon using methanol (29%–55%) instead of acetonitrile.

### Selectivity, precision and performance parameters

**Figure 2.**
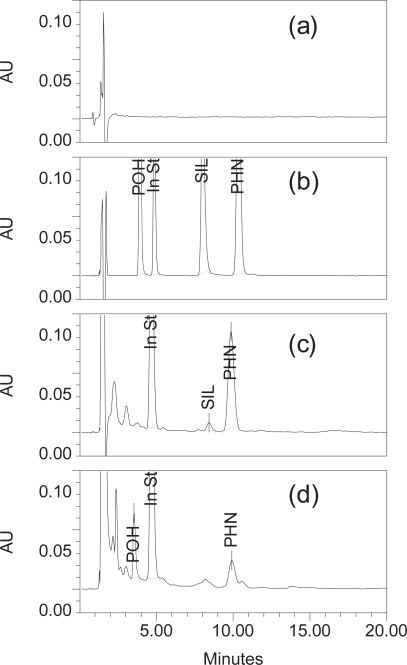
Representative chromatograms of blank plasma extract (**a**), standard calibration mixture (**b**)*, and rabbit plasma extract after 2 hours (**c**)^†^ and after 12 hours from receiving PHN and SIL (**d**)^‡^. (PHN at 10.12 min, POH at 3.83 min, SIL at 8.29 min, and In St at 4.76 min). *POH, SIL and PHN amounts, 15, 20 and 25 ng/μl, respectively. ^†^SIL and PHN amounts, 0.19 and 14 μg/ml, respectively. ^‡^POH and PHN amounts, 3.6 and 2.2 μg/ml, respectively.

Upon application of the chromatographic conditions mentioned in the experimental part, a complete separation between all investigated substances and biogenic plasma constituents was observed (Fig. 2). The chromatographic performance parameters of the drugs extracted from rabbit plasma are shown in Table 1. The RSD values of all chromatographic parameters were not more than 1.5%.

### Linearity and range

A linear HPLC response of percentage peak area ratio of drug to internal standard peak areas for PHN, POH, and SIL were observed over the range, 0.15–39, 0.15–33, and 0.15–34 μg/ml, respectively. The calibration parameters of the investigated compounds in rabbit plasma are listed in Table 2. The values of RSD of limit of quantitation (LOQ) and detection (LOD) were relatively high but within the acceptable range as per the Food and Drug Administration guidelines (did not exceed 20%) (FDA, 2001).

### Precision and accuracy

Within- and between-day precision and accuracy were evaluated by analyzing six replicates of quality control samples at four different concentrations of PHN, POH, and SIL (Table 3). Precision was expressed as the coefficient of variation, though accuracy was presented as a percent error (relative error), [(observed concentration—nominal concentration)/nominal concentration] × 100]. Within- and between-day relative standard deviations were less than 3.0%. Accuracy was within 7.2% for low concentrations and 2.0% for high concentration levels when compared with nominal concentrations. The results indicate that the method is reliable, reproducible, and accurate.

### Recovery and stability (QC samples)

The precisions of % recoveries of PHN, POH, and SIL from plasma were calculated from the corresponding calibration curve of each. The % recoveries of PHN, POH, and SIL from plasma were, 101.88 ± 0.12% (n = 6; RSD = 0.11%), 99.16 ± 0.25% (n = 6; RSD = 0.29%), 99.49 ± 0.33% (n = 6; RSD = 0.33%), respectively. The recovery of POH was relatively low; however, it was enhanced by reducing the wash volume from 20 ml to 3 ml. Also, the spiking volume was considered. About 84% of SIL was recovered from plasma upon using diethyl ether and trifluoroacetic acid, however, complete recovery was obtained upon using ethyl acetate alkalinized with diethylamine. Within and between day precision and accuracy data for determination of PHN, POH, and SIL in spiked plasma are listed in Table 3.

The stability of the investigated substances in spiked rabbit plasma was investigated through three freeze—thaw cycles of the QC samples during the storing period of 0, 10, 30 days at −20 °C. All investigated substances were considered stable in rabbit plasma after three freeze—thaw cycles at nominated concentrations. 101.2% (n = 6; RSD = 0.10%), 99.4% (n = 6; RSD = 0.27%), 99.1% (n = 6; RSD = 0.34%), of PHN, POH, and SIL, respectively. Results of the stability experiments indicated that investigated substances in the plasma samples were stable for at least 1 month when stored at −20 °C.

### Determination of phenytoin in rabbit plasma samples

**Figure 3.**
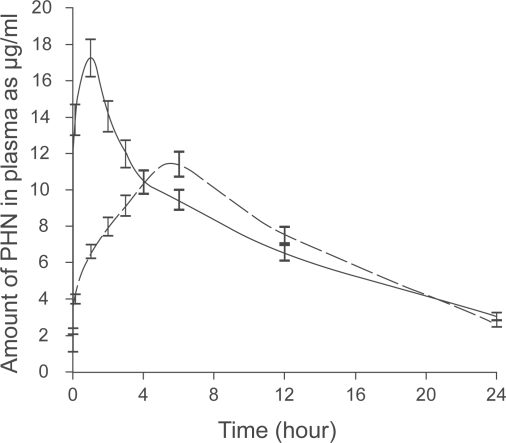
Phenytoin plasma concentration versus time determined following receiving 30 mg/kg of PHN (____) or 30 mg/kg PHN with 8 mg/kg SIL (– – –) (average data of 9 animals).

The developed method of analysis was applied for determination of PHN in rabbit plasma in presence and absence of SIL. No interference from endogenous plasma substances, SIL or POH. The amounts of PHN recovered (as μg/ml) from rabbit plasma were plotted versus time in hours. Figure 3 shows the PHN plasma curve in both cases. The pharmacokinetic profile of PHN was not calculated due to small number of animals used. However, this method could be applied for the study of the impact of SIL on the pharmacokinetic of PHN using large number of animals and suitable pharmacokinetic statistical model.

## Conclusion

The described HPLC method is selective, precise, and sufficiently suitable for the analysis of PHN in presence of SIL, in rabbit plasma. The extraction procedure is characterized by its precision and high recovery yield of PHN, POH, and SIL using two step extractions on Sep-Pak C_18_ columns. Samples could be analyzed by HPLC within only 15-min run time. The developed method could be applied for the study of the impact of SIL on the pharmacokinetic of PHN on epileptic patients.

## Figures and Tables

**Figure 1.**
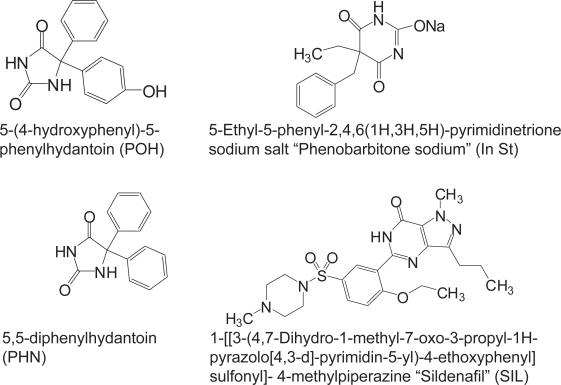
Chemical structure of the investigated substances.

**Table 1.** The chromatographic performance parameters* of the drugs extracted from rabbit plasma using Sep-Pak C_**18**_.

**Name**	**RT (min)**	**Area† (RSD)**	**Area (%)**	**Width (sec)**	**K’**	**α**	**R (RSD)**	**As**	**N**
POH	3.913	2807047 (0.23)	1.68	0.88	2.91			1.31	4522
In St	4.857	1668278 (0.03)	–	0.83	3.86	1.32	3.87 (0.03)	1.24	6365
SIL	8.007	2538179 (0.12)	1.52	1.58	7.01	1.82	8.96 (0.10)	1.48	5203
PHN	10.346	3960490 (0.07)	2.37	1.17	9.35	1.33	5.24 (0.01)	1.13	9309

* K’ capacity factor; α, selectivity coefficient; R, USP resolution; and As, peak asymmetry; N, USP plate count. All parameters were calculated as per United States Pharmacopoeia.

^†^ (Concentrations, 0.36, 0.26 and 0.23 μg/ml of PHN, POH and SIL, respectively).

**Table 2.** Calibration parameters of the investigated compounds in rabbit plasma.

**Name**	**Calibration range (μg/ml)**	**LOQ as μg/ml (RSD)**	**LOD as μg/ml (RSD)**	**Slope^†^**	**Intercept^†^**	**R^2†^**
PHN	0.15–39	0.15 (7.4)	0.050 (8.3)	2.180	0.950	0.998
POH	0.15–33	0.15 (7.6)	0.050 (7.8)	2.672	−0.300	0.997
SIL	0.15–33	0.15 (9.3)	0.005 (12.3)	2.680	0.278	0.999

^†^ Calibration parameters calculated as a function of x = amount of PHN as ng/μl of the injected solution, and Y = % of peak area ratio of PHN to In St. The x values were multiplied by 1.5 to get the drug amount as μg/ml.

**Table 3.** Within and between day precision and accuracy for determination of PHN, POH and SIL in spiked rabbit plasma.

**Nominal concentration (μg/ml)**		**Within – day**			**Between – day**		
		Observed Concentration* as μg/ml ±SD	**CV† (%)**	**Relative error (%)**	Observed Concentration* as μg/ml ±SD	**CV† (%)**	**Relative error (%)**
PHN	3.00	2.97 ± 0.07	2.35	1.01	3.06 ± 0.04	1.31	1.96
	6.00	5.95 ± 0.12	2.02	0.84	5.97 ± 0.11	1.84	0.50
	18.00	17.84 ± 0.47	2.63	0.90	17.88 ± 0.50	2.79	0.67
	39.00	38.25 ± 1.05	2.74	1.96	38.32 ± 0.96	2.51	1.77
POH	3.00	2.74 ± 0.08	2.86	7.14	2.84 ± 0.08	2.82	5.63
	6.00	5.79 ± 0.11	1.90	3.63	5.74 ± 0.11	1.92	4.53
	18.00	17.90 ± 0.50	2.79	0.56	17.68 ± 0.50	2.83	1.81
	33.00	31.81 ± 0.81	2.55	3.74	32.50 ± 0.81	2.49	1.54
SIL	3.00	3.11 ± 0.08	2.57	3.54	2.89 ± 0.08	2.77	3.81
	6.00	5.81 ± 0.07	1.20	3.27	5.94 ± 0.07	1.18	1.01
	18.00	17.74 ± 0.71	2.31	1.47	17.66 ± 0.41	2.32	1.93
	33.00	32.60 ± 0.72	2.21	1.23	32.80 ± 0.72	2.20	0.61

* Mean (standard deviation), n = 6.

^†^ CV = coefficient of variation.
